# Cryo-EM enters a new era

**DOI:** 10.7554/eLife.03678

**Published:** 2014-08-13

**Authors:** Werner Kühlbrandt

**Affiliations:** 1 **Werner Kühlbrandt** is in the Department of Structural Biology, Max Planck Institute of Biophysics, Frankfurt, Germanywerner.kuehlbrandt@biophys.mpg.de

**Keywords:** Cryo-EM, image analysis, single-particle analysis, micoscopy, *E. coli*, human, *S. cerevisiae*

## Abstract

Advances in detector hardware and image-processing software have led to a revolution in the use of electron cryo-microscopy to determine complex molecular structures at high resolution.

**Related research article** Scheres SHW. 2014. Beam-induced motion correction for sub-megadalton cryo-EM particles. *eLife* **3**:e03665. doi: 10.7554/eLife.03665

**Image** Electron micrograph of showing particles of the enzyme γ-secretase

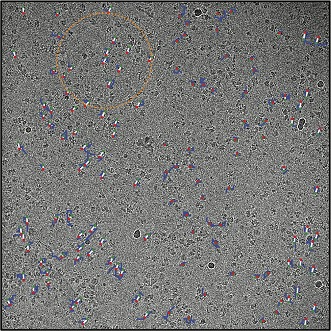

The development of new detector hardware has led to a resolution revolution in electron cryo-microscopy (cryo-EM; Kühlbrandt, 2014). This is evident from a series of striking new structures obtained by cryo-EM at near-atomic resolution: these include ribosomes from human pathogens (Wong et al., 2014) or mitochondria (Amunts et al., 2014), ribosomes in complex with a protein translocase (Voorhees et al., 2014), ion channels (Liao et al., 2013; Cao et al., 2013), or a key enzyme in the biogenesis of methane (Allegretti et al., 2014). The ability to solve such structures in atomic detail is an essential prerequisite for the development of novel antibiotics and drugs.

The complexes mentioned above are mostly in the megadalton size range, which has long been the domain of cryo-EM. Recently, however, a team of researchers led by Sjors Scheres of the MRC Laboratory of Molecular Biology (LMB) and Yigong Shi of Tsinghua University showed that cryo-EM can also be used for smaller, asymmetrical membrane proteins (Lu et al., 2014). They did this by working out the structure of an enzyme called the human γ-secretase complex at a resolution of 4.5 Å: this enzyme cleaves a variety of proteins within cells, and if it does not work properly, the end result can be Alzheimer's disease. The ordered regions of this complex have a molecular mass of 170 kilodaltons (kDa), which was previously thought to be too small for cryo-EM, even when the latest direct electron detectors were being used. Now, in *eLife*, Scheres describes the methods that have made this breakthrough possible (Scheres, 2014).

A major advantage of cryo-EM over X-ray crystallography is that the molecule of interest does not have to be crystallized. Instead, a thin film (ideally not much thicker than the molecule itself) of an aqueous solution is rapidly frozen on a support grid—so rapidly that the water has no time to crystallize. The vitrified sample is then placed in the high vacuum of the electron microscope, where it is cooled with liquid nitrogen to −180°C. Projection images of multiple copies of the molecule, suspended in random orientations in the vitrified film, are recorded with a beam of 200 kV or 300 kV electrons, and the structure of the molecule is determined by combining the projection images into a 3D molecular volume in a computer.

Because the information in each image is noisy and incomplete, tens or hundreds of thousands of particle images are aligned and averaged. The quality of the final map of the molecule depends on the accuracy to which the particle images can be aligned. This works better for larger particles that are more easily visible, and therefore there is a lower size limit in cryo-EM, which until now was thought to be around 300 kDa. However, it should be noted that, with perfect images, theoretical considerations (Henderson, 1995) indicate a lower size limit of 38 kDa.

The main challenge in cryo-EM is low signal-to-noise ratio or, in other words, poor image contrast. Low contrast is the inevitable consequence of the fact that all biological objects and their components—proteins, nucleic acids, carbohydrates, lipids—are radiation-sensitive. In order to determine their structures in atomic detail, as is necessary to understand how they function in the cell, only very low electron doses can be applied. Exposures above a critical limit break covalent bonds irreversibly, destroying the very detail we want to know about.

To make matters worse, the particles must not move by more than about 1 Å, which is equivalent to the diameter of one hydrogen atom, while an image is being recorded. However, much larger movements usually occur when the electron beam hits a biological specimen, possibly due to the release of stresses that result from the sample and the gird and film that support it all having different thermal expansion coefficients. Electron irradiation breaks covalent bonds and creates gas molecules (such as hydrogen, oxygen, nitrogen and methane) that push through the ice to escape. These unavoidable beam-induced movements have made it extremely difficult to collect high-resolution electron micrographs of biological macromolecules in the past.

It takes seconds to record a low-dose image on conventional media (CCD cameras or photographic film), during which time small movements build up and blur the high-resolution features. The new direct electron detectors are both more sensitive and much faster than conventional media. Instead of a single, second-long exposure, they record movies at a rate of many frames per second. Successive movie frames are compared in order to detect any movements on the Å scale. Once the movements of the particles have been traced, they can be reversed in the computer. The movie frames are then added up to give one much sharper, motion-corrected image.

To take full advantage of the new detector hardware, programs were developed to perform movie processing and motion correction (Bai et al., 2013; Li et al., 2013) and, above all, to extract the precious structural information from noisy images in an optimal way. A program suite based on maximum likelihood and prior knowledge (such as a reference volume) about the structure to be determined (Scheres, 2012) has taken the field by storm. The new software works largely without user intervention and provides objective resolution criteria that have quickly become the ‘gold standard’ in cryo-EM. Standardized validation procedures make results more reliable (Rosenthal and Henderson, 2003) and produce clearer maps by helping to avoid over-fitted noise (Scheres and Chen, 2012) that has bedeviled many of the previously published single-particle cryo-EM structures. Powerful classification schemes separate different molecules (Amunts et al., 2014) or distinct structural states of the same molecule (Fernandez et al., 2013) that may be simultaneously present in one field of view. In effect, the sample is purified in the computer rather than by arduous, time-consuming and often damaging biochemical procedures.

For large assemblies, such as ribosomes, movements are corrected for each particle individually, but this is not feasible for small, low-contrast proteins. Instead, Scheres has now extended his original procedure to include statistical movie processing of an ensemble of particles and to take beam-induced motion and radiation damage into account. This has resulted in the structure of the γ-secretase complex at 4.5 Å resolution (from 144,000 particles), and also in significant improvements of several other structures. The 4.5 Å map reveals the secondary structure of the complex, both in the cytoplasmic domain and in the membrane, where the active site resides (Lu et al., 2014; Figure 1).

**Figure 1.**
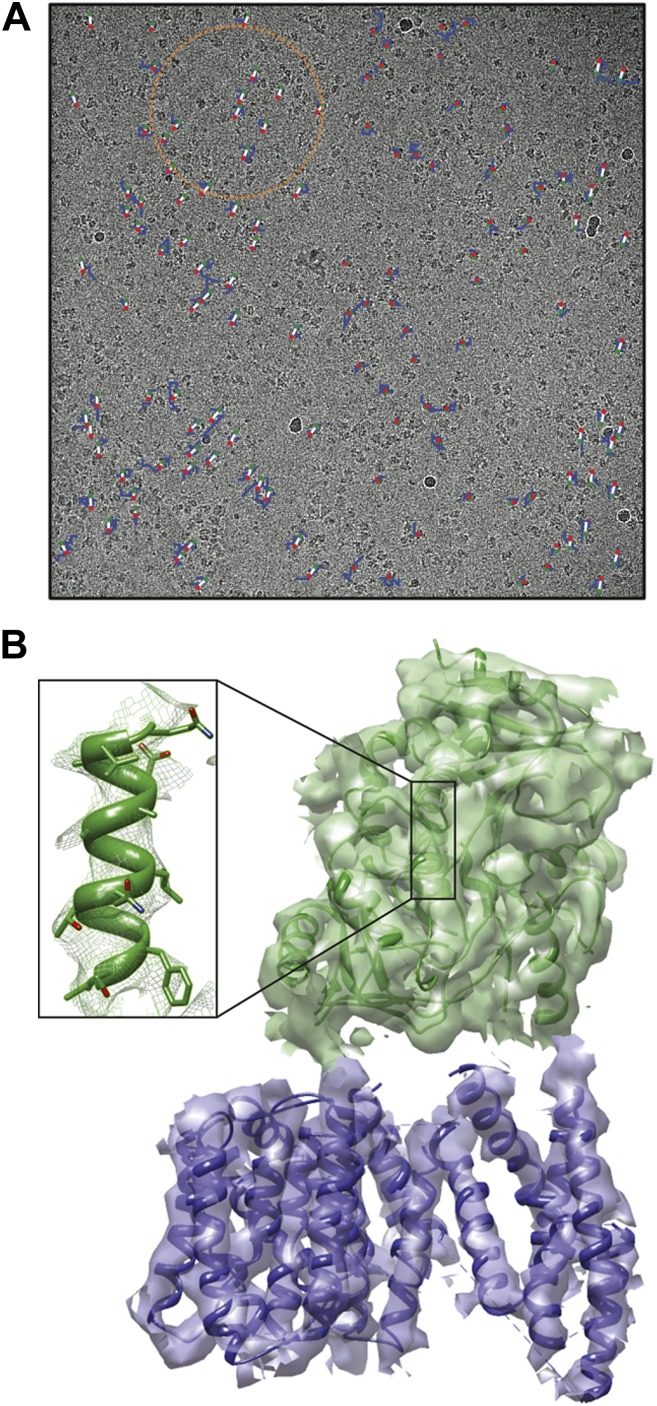
Cryo-EM structure of the human γ-secretase complex. (**A**) Electron micrograph showing how the γ-secretase particles are moved by the electron beam (blue tracks; movement multiplied by a factor of 50 to make it visible). Scheres and co-workers have developed techniques (Bai et al., 2013; Scheres, 2014) to correct for these movements, and used them to determine the structure of the human γ-secretase complex at a resolution of 4.5 Å (Lu et al., 2014). This approach involves fitting linear tracks to the real movements: the fitted tracks are shown in white, with their start and end points being shown in green and red, respectively; the orange circle outlines the ensemble of particles used for statistical processing to fit the track of one particle (Scheres, 2014). (**B**) 3D map of the γ-secretase complex. The 19 trans-membrane helices of the four subunits that contain the active site of the complex are shown in blue, and the extracellular domain is shown in green. The inset shows an alpha helix with partly resolved sidechains in the extracellular domain.

CREDIT Scheres.

While this result is unprecedented and exciting, the structure-based development of inhibitors that might delay the onset of Alzheimer's disease will require the amino acid sidechains in the active site of the complex to be resolved at 3 Å or better. For the time being, this remains unattainable for such small protein assemblies, even with the latest detector technology and software, but further improvements are on the horizon. These include thinner detector chips and faster readout rates, which will greatly enhance the signal-to-noise ratio at high resolution, improvements in specimen preparation, the use of supports that are less prone to move under electron bombardment, and new image recording procedures that reduce beam-induced movement.

Once all these developments are in place, the 20-year old dream of being able to use cryo-EM to determine atomic-resolution structures from just a few thousand particles (Henderson, 1995) may finally come true. Until then, the combination of direct electron detectors and reliable image processing programs is keeping the field moving forward at a rapid rate.
